# Disordered Eating Attitudes, Anxiety, Self-Esteem and Perfectionism in Young Athletes and Non-Athletes

**DOI:** 10.3390/ijerph17186754

**Published:** 2020-09-16

**Authors:** Cristina Petisco-Rodríguez, Laura C. Sánchez-Sánchez, Rubén Fernández-García, Javier Sánchez-Sánchez, José Manuel García-Montes

**Affiliations:** 1 Research Group Planning and Assessment of Training and Athletic Performance, Faculty of Education, Pontifical University of Salamanca, Calle Henry Collet, 52-70, 37007 Salamanca, Spain; cpetiscoro@upsa.es (C.P.-R.); jsanchezsa@upsa.es (J.S.-S.); 2 Department of Evolutionary and Educational Psychology, Faculty of Science Education and Sport, University of Granada, Calle Santander, N° 1, 52071 Melilla, Spain; 3 Department of Nursing, Physiotherapy and Medicine, University of Almeria, Carretera Sacramento, S/N, La Cañada de San Urbano, 04120 Almería, Spain; rubenfer@ual.es; 4 Department of Psychology, University of Almeria, Carretera Sacramento, S/N, La Cañada de San Urbano, 04120 Almería, Spain; jgmontes@ual.es

**Keywords:** eating disorders, prevention, psychological factors, athletes, non-athletes, gymnasts, footballers

## Abstract

Eating disorders are associated with short and long-term consequences that can affect sports performance. The purposes of this study were to investigate whether female athletes, particularly gymnasts and footballers, exhibit more eating problems compared to female non-athletes, and to identify individual personality characteristics including anxiety, self-esteem, and perfectionism as possible contributors to eating disorder risk. In a sample of 120 participants, 80 adolescent female athletes were compared to a control condition of 40 non-athletes (mean age 17.2 ± 2.82). Participants responded to a questionnaire package to investigate the presence of disordered eating (SCOFF) and psychological variables in relation to disordered eating symptoms or eating disorder status. Subsequently, anthropometric measures were obtained individually by trained staff. There were statistically significant differences between conditions. One of the most important results was the score in SCOFF (Mann–Whitney = 604, *p* < 0.05; Cohen’s *d* = 0.52, r = 0.25), being higher in control than in the gymnast condition. These results suggest that non-athlete female adolescents show more disturbed eating behaviours and thoughts than female adolescents from aesthetic sport modalities and, therefore, may have an enhanced risk of developing clinical eating disorders.

## 1. Introduction

Eating disorders (EDs) are common and potentially serious conditions that have devastating effects on physical/emotional health, sports performance, and overall quality of life, carrying an increased risk of morbidity and mortality [1,2]. The Diagnostic and Statistical Manual of Mental Disorders (DSM-5) categorizes EDs into several specific types, including: Anorexia Nervosa (AN), Bulimia Nervosa (BN), Binge Eating Disorder (BED) Pica, Rumination Disorder, Avoidant/Restrictive Food Intake Disorder (ARFID), Other Specified Feeding or Eating Disorder (OSFED), Other Specified Feeding or Eating Disorder (OSFED), and Unspecified Feeding or Eating Disorder (UFED) [3].

Identification of risk factors is needed to best target prevention and intervention strategies for vulnerable populations [4]. In sport, there is often an ideal body image that suggests improved performance, and this may be one of the reasons why athletes are at increased risk of disordered eating [5], especially in endurance, aesthetic and weight class sports that emphasize leanness or a low body weight [6,7]. Besides, other factors such as the pressure from coaches, parents, and other participants to lose weight for competition, dieting, frequent weight cycling, early start of sport-specific training, over-training, injuries, or the personality characteristics of athletes, may account for athlete’s propensity for eating disorders [8,9]. Teammates are also an important source of influence on athlete eating attitudes and behaviours and these influences can be protective against or engender an increased risk for disordered eating [10].

The risk of developing EDs among athletes varies, depending on gender, sports discipline and competitive level [11]. In order to enable elite athletes to perform to their highest potential while maintaining their health, knowledge of the risk of eating disorders in sports is important [12]. Coaches should be aware of this lack of knowledge and work with clinical practitioners, such as dieticians, team physicians and athletic trainers to educate and monitor the eating disorders of athletes specifically for signs and symptoms [13].

Clinical-level EDs affect women at higher rates than men [14,15]. Eating disorders are particularly common in female adolescents and young adults (90% of cases occur in people under the age of 25) and seem to be more prevalent among athletes than in the general population [16,17]. According to a systematic review that analysed 169 studies, the prevalence of disordered eating ranged from 0 to 27% in female athletes and from 0 to 21% in the general population [18]. Large-scale studies from Norway revealed EDs prevalence rates for female athletes of 42% in aesthetic sports, 24% in endurance sports, 17% in technical sports, and 16% for ball game sports [19]. Similarly, another study examined the ED prevalence rates of German female professional athletes and non-athletes. In this study, the athletes achieved the highest rate of ED (17%) for aesthetic sports, followed by ball game sports (3%), and non-athletes showed the lowest rate (2%) [20]. In the same way, other authors affirmed that gymnasts are frequently subject to constant stress and pressure attributed to their coaches to lose weight, emphasizing unhealthy weight control practices [21].

However, it is unclear whether athletes represent a subgroup that is truly “at risk” of experiencing an eating disorder [22]. A meta-analysis [7] did not find clear evidence for an increased risk of EDs among athletes and even some investigations have found higher frequencies of risky eating behaviours in the general population or non-athletes compared with athletes [23,24,25].

The etiology of EDs is multifactorial and can frequently be attributed to low self-esteem, a distorted body image in which the body is perceived with excess weight, inefficiency, perfectionism, anxiety, emotion dysregulation, goal-orientation, concern with performance, and a sense of control loss [26,27,28]. With respect to personality characteristics, many of the traits considered desirable in elite athletes, such as perfectionism, overlap with those found in eating disordered individuals [29]. Research has demonstrated a relationship between perfectionism and many different forms of anxiety [30] and between anxiety and eating disorders, so that individuals with eating disorders may be linked to other relevant emotions such as anxiety and anxiety sensibility [31]. However, perfectionism is a multidimensional characteristic and only some dimensions of perfectionism are clearly maladaptive in sports, whereas others are not [32]. Anxiety is one of the most common emotional responses in athletes, which has evident consequences on performance [33]. Other well-known risks of eating disorders are low self-esteem and high perfectionism [34]. Athletes with a high self-esteem based on a respect and love for themselves had more positive patterns of perfectionism, whereas athletes who have a self-esteem that is dependent on competence aspects showed a more negative perfectionism [35]. Thus, low self-esteem also appears to be an important risk factor for body image dissatisfaction and eating disturbance, although it is difficult to know whether this is really a cause or an effect [36,37]. According to Fairburn’s transdiagnostic model of EDs, high levels of perfectionism and low self-esteem are two core traits across EDs [36]. Various studies have shown, indeed, that high perfectionism and low self-esteem are both predictors of AN [38,39] and BN [38,40]. However, a recent research was not able to detect these results for all separate ED categories [41].

The serious consequences of EDs and the contradictory results present in the literature in this field, support the need to continue investigating their prevalence among athletes and non-athletes and the risk factors associated with the physique and personality of the individuals. For these reasons, the aims of the current study are: (a) to explore the relevance of physical variables, especially BMI; (b) to examine the prevalence of disordered eating behaviour and attitudes among 80 female athletes (40 gymnasts and 40 football players) and 40 female non-athletes; (c) to investigate the relationships between eating attitudes and physical and psychological dimensions, such as perfectionism, anxiety and self-esteem; and (d) to know the differences between groups in physical and psychological variables.

## 2. Materials and Methods

### 2.1. Participants

The participant sample consisted of 120 Spanish female professional athletes and non-athletes aged 15 to 25 years old who voluntarily agreed to take part in the study. The entire research population was divided into three groups of 40 subjects each: athletes competing in rhythmic gymnastics, athletes competing in football and the control group which consisted of students with a sedentary life style.

With respect to the academic background of the participants, 61 participants studied at secondary school, 35 participants studied sixth form and 24 were higher education students.

The athletes were recruited from six different sports institutions (four elite rhythmic gymnastics schools and two football clubs of national league-level) with assistance from their coaches. The group of gymnasts consisted of five elites competing internationally and 35 gymnasts competing at the national level of competition; the elites spent 20–35 h on training and competition per week and the gymnasts who competed in national tournaments spent 12–20 h on gymnastics and other sport-activities together. The group of footballers belonged to two Spanish teams, and only competed nationally. The regular training schedule of footballers involved four training sessions plus a competitive match per week in the Spanish first and second national female football division “Group V”. Before contacting these athletes, the objectives and procedures of recruitment were explained at the administrative boards of eight clubs and finally, representatives of six clubs agreed to participate, thereby allowing data collection with regard to their athletes. The control group (non-athletes) were recruited among a random sample of students from four different secondary schools and colleges from public and private educational institutions in Spain; none of them were engaged in any competitive sports, nor did they train for a particular type of sport. The inclusion criteria for the athlete groups were membership in Spanish sport clubs of rhythmic gymnastics, or footballers with a background of ≥4 years of systematic training and competitive experience, training a minimum of 8 h per week, with absence of severe injuries (low-risk stress fractures are not included). The exclusion criteria were either lack of consent to participate in the study or failing to complete the questionnaires.

### 2.2. Instruments and Measurements

The evaluation protocol consisted of five questionnaires, which were completed voluntarily, anonymously, individually, and confidentially by the participants, to analyse self-esteem, perfectionism, anxiety and the risk of developing eating disorders. All the final participants filled out the questionnaires in the following order: The Rosenberg Self-Esteem Scale (RSE); The Child and Adolescent Perfectionism Scale (CAPS); The State-Trait Anxiety Inventory (STAI); Eating Attitudes Test (EAT-40); and Sick, Control, One, Fat, Food questionnaire (SCOFF). These questionnaires were composed of a general part concerning the socio-demographic, lifestyle and health conditions (age; education/level of studies; physical activity; and background of cancer, neurological disease, anxiety, depression or eating disorders).

The RSE is a 10-item self-report unidimensional measure of global self-esteem, developed by Rosenberg [42]. The items, divided into five positive and five negative statements, are answered on a four-point Likert scale ranging from 1 (strongly disagree) to 4 (strongly agree). The Spanish adaptation of the Rosenberg Self-Esteem Scale validated in the adolescent population [43], was used to assess global self-esteem. These authors understand self-esteem as a feeling of self, which can be positive or negative, constructed by an evaluation of one’s own characteristics. The Spanish version has a high internal consistency (Cronbach’s alpha between 0.80 and 0.87) and a test-retest reliability of 0.72. The scale ranges from 10 to 40. Scores between 20 and 30 are within the normal range, while scores below 20 suggest low self-esteem and scores higher than 40 suggest high self-esteem.

The CAPS is a self-report questionnaire of 22 items based on a multidimensional conceptualization of perfectionism [44]. It has two scales: Self-Oriented perfectionism (Subscale 1, from here on out, Sub1) with 12 items and Socially-Prescribed perfectionism (Subscale 2, Sub2) with 10 items. Some authors described what they termed self-oriented perfectionism as critical self-scrutiny, unrealistic self-imposed personal standards, and requiring perfection of oneself, and socially-prescribed perfectionism as perceiving that others are demanding perfection of oneself and the need to achieve standards and goals indicated by others [45]. The Spanish version of this scale [46] was used in the study, this version has an α coefficient reliability of 0.91 and a good test-retest reliability.

The STAI is a self-report questionnaire appropriate for measuring anxiety, one of the psychological problems with the highest prevalence [47]. The Spanish adaptation used in this study [48] obtained good reliability indices in psychometric studies and high internal consistency (Cronbach’s alpha between 0.9 and 0.93 in state anxiety and between 0.84 and 0.87 in trait anxiety). This questionnaire assesses Trait Anxiety (understood as a personality factor that predisposes one to suffer from anxiety) and State Anxiety (refers to environment factors that protect from or generate anxiety). Each of the STAI scales (trait anxiety and state anxiety, SATAI-T and STAI-S, from here on out) is composed of 20 items. The response scale is Likert, scoring from 0 (nothing) to 3 (much). Totals are obtained by adding the values of the items (after reversing the scores on the negative items). Therefore, trait anxiety and state anxiety totals range from 0 to 60, corresponding to a higher score with greater anxiety detected [49].

The EAT-40 is one of the most widely used measures in the field of eating disorders, in both clinical and epidemiological studies. It was developed to assess a range of behaviours and attitudes about food, weight and exercise related to anorexia [50]. The EAT-40 is validated in Spain and is the version used in this study [51]. It consists of a self-administered questionnaire of 40 items. Each item is a 6-point Likert scale ranging from “never” to “always”, providing a valid instrument to start the diagnosis and detect incipient cases of eating disorders that have not yet been diagnosed. Twenty-six of the items make up the following three subscales: (1) Dieting—the avoidance of fatty foods and a fixation on losing weight (Subscale 1, Sub1, from here on out); (2) bulimia and food fixation or an indication of bulimia (Subscale 2, Sub2); and (3) the perceived external pressure to gain weight and self-control over eating (Subscale 3, Sub3). The range of scores varies from 0 to 120, where the higher the score, the higher the risk of developing eating disorders. The cut-off point proposed in the original version of 1979 is 30 for a sensitivity of 100% and a specificity of 93%. The Spanish version [51] proposes a cut-off point of 20 to reach a sensitivity of 91% and a specificity of 69%; this version has an α coefficient reliability of 0.93 and a good discriminate validity.

The SCOFF questionnaire consists of five eating-related questions and is effective as a screening instrument for detecting eating disorders. It is simple and easy to apply and score and is designed to raise suspicion of a likely case rather than a diagnosis [52]. Answers (yes/no) are scored by one point for a positive answer and a zero point for a negative answer. Two positive answers in the five SCOFF questions indicate disordered eating behaviour and attitudes [53]. The SCOFF appears to be effective as a screening instrument, with a suitable sensitivity and acceptable specificity to prompt further evaluation and if appropriate medical or psychological diagnosis [54]. This questionnaire has a Cronbach’s alpha of 0.48 and a concordance test re-test of 91.6%. The levels of sensitivity and specificity compared to clinical interviews were 84.6% and 89.6%, respectively [55].

To assess the anthropometric measurements, body mass (kg) and height were measured using the TANITA BC-418MA^®^ body composition analyser scale (0–150 kg, precision 100 g) and Holtex^®^ height rod (60–200 cm, precision 1 mm), respectively. Then, the Body Mass Index (BMI) was calculated (weight in kg/height^2^ in m).

### 2.3. Procedure

The data was acquired through surveys from January to April 2018. The researchers contacted, via email or by telephone, the different technical departments of each of the participating clubs and with the directors of secondary schools to request the authorization and participation of students and athletes who met the inclusion criteria. The researchers informed the contacts about the main objectives of the study. Participation in the study was voluntary, and the young females received no compensation of any kind. All participants, or their parents/legal guardians for those under 18, signed consent forms and received full verbal and written explanation of the purpose of the study, its anonymous nature, and of their ability to withdraw from the experiment at any time. They also received prior instructions on how to fill in the questionnaires and were able to clarify if any doubts arose in the presence of a researcher. In addition, the participants were asked to respond honestly to all the items of the questionnaires. The study was conducted in accordance with the Declaration of Helsinki, and the protocol was approved by the Ethics Committee of the Pontifical University of Salamanca (Acta 20/7/2020).

A total of 65 questionnaires were distributed to each of the different groups of the study (control, gymnasts and footballers), but only 40 were returned properly and entirely completed. The sample finally included 120 participants. The athletes fulfilled the questionnaires after a training session in the facilities where they normally developed their sports practice under the authorization of the sports clubs. Controls filled in the questionnaires during school hours under the authorization of the corresponding teacher and School Board. It took the participants approximately 30 min to complete all the questionnaires. Then, all participants were measured and weighed in private by research staff wearing light clothing and without footwear.

### 2.4. Statistical Analysis

Analysis was performed using SPSS software, version 24 (SPSS, Evanstron, IL, USA). Descriptive statistics were calculated (mean and standard deviation) of the different variables studied. Kolmogorov–Smirnov test was carried out with every variable of the study. The results showed that the variables do not follow the normal distribution of the data (*p* < 0.05). For this reason, non-parametric tests were performed, specifically, the Kruskal–Wallis and Mann–Whitney tests. Subsequently, Cohen’s effect size was calculated in case of statistically significant results. Spearman’s Rank Order correlation coefficient was used for testing possible correlations among the different variables. Finally, multiple linear regression was calculated for SCOFF in order to clarify which variables could most influence on this score.

## 3. Results

### 3.1. Descriptive Analysis: Physical Variables

Firstly, descriptive analysis was carried out, specifically, mean and SD of the health variables (Table 1). The BMI varied between 16.19 and 27.01 (M = 20.63; SD = 2.77). Body Mass Index serves as an indication for low body weight in eating disorders [56]. The vast majority of the sample (*n* = 75, 62.5%) had normal weight (BMI between 18.5 and 24.9), 33 participants (27.5%) were underweight (BMI < 18.5) and the remaining 12 participants (10%) were overweight (BMI > 24.9), according to the classification proposed by the Spanish Society for the Study of Obesity [57].

The main comparison between the groups will be displayed in subsequent analysis.

### 3.2. Prevalence of Disordered Eating Behaviour and Attitudes

In our study, we found that 2.5% of the gymnastics, 12.5% of the footballers and 20% of the non-athletes demonstrated eating attitudes (EAT-40 ≥ 20). The results using the SCOFF questionnaire while applying a cut-off of two positive questions or more to predict the possible presence of an eating disorder, revealed 2/40 (5%), for gymnasts and footballers, and 6/40 (15%) for controls.

Secondly, a multiple linear regression analysis was performed with SCOFF scores as dependent variable and age, weight, height, BMI, and scores in every scale and subscale (self-esteem, perfectionism, anxiety) as independent variables, in order to explore which variables have the most predictive value on the SCOFF scores, that is, forecasting eating disorders. The most predictive variable was Socially prescribed perfectionism (*p* < 0.04) (Table 2).

### 3.3. Relationships between Eating Attitudes and Physical and Psychological Dimensions

Subsequently, correlational analysis with Spearman test was applied in order to explore the relations between the main variables. BMI had a statistically significant correlation with weight. Self-esteem global had a statistically significant negative correlation with weight, perfectionism subscale (CAPS, Sub2); with anxiety subscales (STAI-S and STAI-T); and with EAT40 (Sub2) and SCOFF; but it had a statistically significant positive correlation with CAPS Sub1. That is: As the score in weight, perfectionism, anxiety, and probability of feed disorder was increasing, the score in self-esteem was decreasing and vice versa. The perfectionism (CAPS) had a statistically significant positive correlation with anxiety (STAI), that is, as the score in perfectionism was increasing, so did the anxiety, and vice versa. Finally, the eating attitude (EAT40, Sub1 and Sub2) had a statistically significant positive correlation with anxiety, that is, as the scores in the subscales of anxiety increased, the eating attitude worsened (Table 3).

### 3.4. Comparison between Conditions

After that, the Kolmogorov–Smirnov test was carried out with each variable of the study. The results showed that the variables do not follow the normal distribution of the data (*p* < 0.05). For this reason, non-parametric tests were performed, specifically, the Kruskal–Wallis and Mann–Whitney tests.

Following that, the Kruskal–Wallis test was applied to all the data in order to compare the group (as factor) in the main variables of the study. The test indicates that there are statistically significant differences between the group in the BMI (χ^2^ = 33.86, *p* < 0.05); in the Rosenberg Self-Esteem Scale (Global score) (χ^2^ = 29.05, *p* < 0.05) and in the two subscales scores of this scale, Positive (χ^2^ = 10.99, *p* < 0.05) and Negative (χ^2^ = 6.30, *p* < 0.05); in Subscale 1 of the CAP (χ^2^ = 11.34, *p* < 0.05); in STAI-Estate (χ^2^ = 5.65, *p* < 0.05); and in SCOFF (χ^2^ = 7.90, *p* < 0.05) (Table 4).

Subsequently, three Mann–Whitney tests were performed in order to clarify between which groups the main differences were. In the comparison between the gymnastics and footballer groups, the statistically significant differences were found in the following variables: in the BMI (Mann–Whitney = 248, *p* < 0.05), being higher in the footballer than in the gymnast condition and there was a high to very high effect size, Cohen’s *d* = 1.59 (r = 0.62); in the Rosenberg Self-Esteem Scale (Global score) (Mann–Whitney = 526, *p* < 0.05), being higher in the gymnast than in the footballer condition and there was a moderate effect size, Cohen’s *d* = 0.57 (r = 0.27); in the Negative subscale (Mann–Whitney = 566, *p* < 0.05), being higher in the gymnast than in the footballer condition and there was a moderate effect size, Cohen’s *d* = 0.46 (r = 0.23); and in STAI-Estate (Mann–Whitney = 579, *p* < 0.05), being higher in the footballer than in gymnast condition and there was a small to moderate effect size, Cohen’s *d* = 0.56 (r = 0.27) (Table 5).

In the comparison between the footballers and control groups, the statistically significant differences were found in the following variables: in the BMI (Mann–Whitney = 512.5, *p* < 0.05), being higher in the footballer than in control condition with a moderate to high effect size, Cohen’s *d* = 0.65 (r = 0.31); in Rosenberg Self-Esteem Scale (Global score) (Mann–Whitney = 492, *p* < 0.00), being higher in the footballer than in the control condition with a very high effect size, Cohen’s *d* = 2.3 (r = 0.75) and marginally significant in the Positive subscale (Mann-Whitney = 607, *p* = 0.06), being higher in the footballer than in control condition and there was a small to moderate to high effect size, Cohen’s *d* = 0.48 (r = 0.23); and in the Subscale 1 of the CAP (Mann–Whitney = 547, *p* < 0.05), being higher in the footballer than in the control condition with a small to moderate to high effect size, Cohen’s *d* = 0.52 (r = 0.25) (Table 5).

In the comparison between the gymnastics and control groups, the statistically significant differences were found in the following variables: in the BMI (Mann–Whitney = 393, *p* < 0.05), being higher in the control than in the gymnast condition and there was a high to very high small effect size, Cohen’s *d* = 1.02 (r = 0.45); in Rosenberg Self-Esteem Scale (Global score) (Mann–Whitney = 256, *p* < 0.05), being higher in the gymnast than in the control condition with an almost perfect effect size, Cohen’s *d* = 3.87 (r = 0.89) as well as in the two subscale scores of this scale, Positive (Mann–Whitney = 458.5, *p* < 0.05), being higher in the gymnast than in the control condition and there was a moderate to high effect size, Cohen’s *d* = 0.81 (r = 0.38), and in the Negative subscale (Mann–Whitney = 589.5, *p* < 0.05), being higher in the gymnast than in the control condition with a small to moderate effect size, Cohen’s *d* = 0.37 (r = 0.19); in Subscale 1 of the CAP (Mann-Whitney = 471, *p* < 0.05), being higher in the gymnast than in the control condition with a moderate to high effect size, Cohen’s *d* = 0.76 (r = 0.35); in STAI-Estate (Mann–Whitney = 596.5, *p* < 0.05); being higher in the control than in the gymnast condition with a small to moderate effect size, Cohen’s *d* = 0.51 (r = 0.25) and marginally significant in STAI-T (Mann–Whitney = 602.5, *p* = 0.06), being higher in the control than in the gymnast condition with a small to moderate effect size, Cohen’s *d* = 0.46 (r = 0.23); in Subscale 1 of the EAT-40 (Mann-Whitney = 580, *p* < 0.05), being higher in the control than in the gymnast condition with a small to moderate effect size, Cohen’s *d* = 0.53 (r = 0.26); and in SCOFF (Mann-Whitney = 604, *p* < 0.05), being higher in the control than in the gymnast condition with a small to moderate effect size, Cohen’s *d* = 0.52 (r = 0.25) (Table 5).

## 4. Discussion

### 4.1. Physical Characteristics of the Sample

The physical characteristics of the participants are presented in Table 1. There were statistically significant differences between the groups in terms of age, weight and BMI.

However, the gymnastic female athletes participating in the present study were younger and had lower weight and height, compared with the footballer and control groups. As expected, ball game sport athletes were older and heavier than aesthetic sports athletes, similar to a previous study [20]. The mean BMI values in all the groups indicated a normal body weight according to the Spanish Society for the Study of Obesity [57]; however, the BMI of the gymnasts (average of 18.7) is close to underweight values. Due to the fact that gymnasts have a high muscle mass relatively to their body mass, it would be expected that they relatively would have higher BMI. Based on this, it could be expected that several of the gymnasts actually are underweight, as BMI is not always accurate for athletes with high muscle mass. There were six cases of participants being underweight in the rhythmic gymnastics group and one case in the football group. In relation to being overweight, the presence of nine cases is highlighted in football and three in the control group. The results of our study did not show a significant relationship between cases of participants being underweight and increased prevalence of risk attitudes to EDs. These results are in accordance with previous findings that do not support the hypothesis that lean athletes exhibit more eating disordered behaviour than non-lean athletes [7,22,58]. However, there was a significant relationship between the risk of EDs and being overweight, not directly but mediated by global self-esteem. That is, weight correlated inversely with Global self-esteem and this last variable correlated inversely with EAT-40 (Subscale 2) and with SCOFF. These results are in line with those of other studies in which rates of disordered eating were highest among overweight participants [53,59]. Nevertheless, some researchers suggested caution in using BMI or weight alone as a criterion to identify athletes with EDs [60]. It is clear that both coaches and athletes alike should be made aware that when athletes are underweight, they should not be training or competing until they restore a healthy body weight [61]. Nevertheless, overweight individuals may be likely to engage in dieting with the intention of losing weight, thus putting themselves at a greater risk for binge eating, a well-established phenomenon in overweight adolescents [53,62]. In this study, the negative correlation found between weight and Global self-esteem is in accordance with other researches [63,64], in fact, being overweight was associated with lower self-esteem. For this reason, self-esteem could be considered a mediating factor for a greater or lower risk of eating disorder, which has been explored by other authors [65].

In relation to age, our sample ranged between 15 and 25 years old, showing a significantly greater risk of developing eating disorders between 15 and 21 years old; in fact, all the positive cases in SCOFF, the screening scale, were between these ages. This finding is in agreement with available research showing that the peak age of highest risk eating disorders seems to be around 16 to 20, approximately when young people are leaving home and starting college [66].

### 4.2. Prevalence of Disordered Eating Behaviour and Attitudes

The results of our study showed higher scores in EAT-40 for controls (eight cases) and female footballer groups (five cases), with respect to rhythmic gymnastics (one case). Likewise, the greatest risk group using SCOFF was the control group (six cases) versus gymnasts and footballers, both with two cases. In fact, there were statistically significant differences between the control and the gymnast condition in SCOFF and in EAT-40 (Subscale 1). In a study analysing eating attitudes in a sample of adolescents from Sevilla (Sevilla, Spain), an adequate concordance was found between both instruments, considering EAT-40 and SCOFF useful for the screening of EDs, although a structured clinical confirmation interview is necessary [67]. However, the SCOFF is characterized by a tendency toward overinclusion [68]. The prevalence of positive SCOFF screening in our study has been lower than that reported in a sample of 3457 students aged 18 to 25, 20.5% [69], and also lower than those obtained among student populations in Greece, reporting a 39.7% of individuals with SCOFF score ≥ 2 [70]. Similar to our results, although obtaining higher percentages of prevalence, using EAT-26 revealed a lower prevalence (18.1%) of unhealthy eating behaviour in female athletes than in female non-athletes who showed a 26.1% risk for developing ED [23].

In previous literature, higher frequencies of risky eating behaviour in the general population compared with athletes have been confirmed by a number of previous studies [23,71,72]. However, other studies have consistently showed a higher prevalence among athletes compared with controls [19,59,73] or no statistically significant difference between athletes and controls [24,74,75]. Therefore, the research has shown contradictory findings with regard to the prevalence of EDs among female athletes and non-athletes. Thus, it is unclear whether athletes represent a subgroup that is truly at risk of experiencing an eating disorder [22]. This could be due to the existing different instruments used to identify ED, the variability among the sports evaluated or the differences in athlete characteristics (age, performance level). As mentioned by other authors [23], standardizing the use of tools to assess the eating behaviours of athletes would be an interesting option to enable future, reliable comparisons within scientific literature. Furthermore, the fact that both athletes and the general population are vulnerable to media messages about body appearance and social acceptance [76] and that, although some components of sport activity may actually protect against the development of EDs, others appear to increase the risk [77], which could condition the disparity found in the investigations. Another circumstance is the fact that adolescents with eating disorders often deny their problem, and completing scales is subject to the bias of social desirability.

Prevalence rates for disordered eating vary depending on the type of sport [19]. In general, the literature associates athletes in endurance, aesthetic and weight class sports, where leanness or a specific weight are believed to favour sports performance, with a greater risk of developing eating disorders than the general population [7,19]. As is concluded in another previous study, female adolescent athletes, especially those in aesthetic sports, are a group at increased risk of the development of eating disorders and should be taken seriously [78]. The low prevalence of disordered eating behaviour in the gymnasts in our study is quite surprising. In this line, other authors also indicated that gymnasts were slightly less likely to show signs of eating problems than non-athletes [7]. Nevertheless, some research on female gymnasts has suggested that these athletes are at a higher risk of developing eating disorders compared with athletes in other sports [59,79]. One study suggested incidence rates of eating disordered behaviours in gymnasts of around 60% [80] while another obtained percentages of 18% [21]. The implementation of nutritional education on healthy eating practices and the prevention techniques recommended by the results of numerous studies, may be working by reducing the incidence of eating disorders in athlete populations, particularly in gymnastics, which would be a high milestone. According to some researchers, the typical focus on sports such as gymnastics, which is considered particularly of high risk in previous studies, may have led to reform this sport, or this attention may have also led coaches and athletes in these sports to be less communicative when reporting eating problems for fear of damaging their sports image [7]. However, the elite rhythmic gymnasts are very thin, almost anorexic-like physique, but without psychological distress because they have a more a precise estimation of their body image than the other sport athletes, and this condition leads to hypothesize that they are not at risk of eating disorders development [58]. Again, the differences found in literature may be attributed to age, individual personality, different sample size or group composition, contextual differences or the varied methods used to assess eating problems [21].

Although our results reflect a lower risk in the athlete population against a non-athlete population, they may still be vulnerable. This is in line with previous findings [22]. Furthermore, it could be hypothesized that several females in our study suffer from ED and do not give honest responses in questionnaires. In this sense, research shows that athletes tend to underreport in questionnaires [19], which calls for the need of diagnostic interviews. In any case, disordered eating must be considered seriously as it can be associated with health risks such as amenorrhea, osteoporosis, and clinical eating disorders [81]. With respect to prevention of EDs, the available literature suggests that selective primary interventions with multiple targets and an interactive multimodal approach appear most effective [76].

### 4.3. Psychological Factors Related to Disordered Eating Attitudes

The predisposition to develop an ED is dependent on sociocultural, demographic, environmental, biological, psychological, and behavioural factors [82]. In this study, we are focused on the relationship between eating disordered behaviour and psychological factors such as anxiety, self-esteem or perfectionism. These factors can affect eating behaviour, sports performance and general health and wellbeing in athletes and non-athletes [83,84]. Correlation obtained between anxiety (both, trait and state) and the EAT-40 was positive and statistically significant in our study. This result is in line with those achieved in a group of taekwondo and judo athletes and non-athletes using EAT-26 and STAI [74]. Similarly, other researchers also revealed that DE attitudes were significantly positively correlated with anxiety levels [24], thus athletes with disordered eating experience have shown higher levels of both state and trait anxiety compared with athletes without disordered eating behaviour [85]. Other research confirms that anxiety is higher in individuals with eating disorder risk [69,86]. Likewise, other studies have found that higher levels of sport anxiety predicted more bulimic symptoms and a high drive for thinness in female athletes [87].

Perfectionism is a personality characteristic that entails a combination of exceedingly high standards and a preoccupation with extreme self-critical evaluation [88]. In some studies, with female athletes, there is no evidence of a strong relationship between perfectionism and disordered eating [89]. Regression analysis revealed in our study that perfectionism was positively correlated with anxiety levels. The previous literature has suggested that the effects of perfectionism may be indirect through an interaction with other psychological risk factors (e.g., body dissatisfaction) [11]. The results of the present study show statistically significant correlation between self-esteem and the two subscales of perfectionism (CAPS). However, these correlations are positive with the first subscale (self-oriented perfectionism) and negative with the second subscale (socially-prescribed perfectionism). These results agree with other authors who showed that the relation between self-esteem and perfectionism differs depending on which dimensions of self-esteem and perfectionism are being considered [35]. It seems likely that high self-esteem is related to high self-oriented perfectionism, but with low socially-prescribed perfectionism, to the extent that a person with positive self-esteem will not mind the opinion of others. In the multiple linear regression analysis that was performed with SCOFF scores as a dependent variable, the most predictive variable was socially-prescribed perfectionism. Other authors have explored this variable as a mediator in eating disorders or have found an indirect or direct relation between perfectionism and eating disorders [30,90].

Regarding self-esteem, it showed statistically negative correlation with EAT-40 (Sub. 2). Self-esteem has been consistently associated with lower levels of eating and body image disturbances [91] because it involves a positive self-evaluation that may protect women through its amelioration of negative affections and reduction in subsequent binge eating [27]. The most curious data in our study is that self-esteem was higher in athletes than in the control group and better in gymnasts than in footballers. These results are in line with other researches [92,93], pointing out the importance of the practice of sport and of the type of sport, because self-esteem is different in aesthetic sports than in non-aesthetic sports, like football. Self-esteem is probably a protective factor to develop an eating disorder despite perfectionism, which is the most predictive variable as the multiple linear regression showed, and this variable was high in athletes. The fact of training, of having to be fit, forces athletes to take care of their body in a healthy way. However, in the control condition, the participants are under the pressure of social aesthetic standards, which are more and more restrictive and they can try to lose weight, to look thinner, but not because they want to have a healthy lifestyle but more for social-aesthetic reasons, for socially-prescribed perfectionism. As a consequence, the control condition showed the lowest self-esteem and the highest risk of eating disorder. In fact, self-esteem has been highlighted as one of the main mediators in the prevention of eating disorders [94].

### 4.4. Limitations and Future Research

The present study has certain limitations that need to be acknowledged. Owing to our rather small sample size, the generalization of our results is further limited. It is relevant to confirm these results in future studies with larger samples and with other athletes, such as males or participants from other sports. It is also interesting to carry out long-term follow-up studies to control whether these results are maintained or modified over the years. In addition, it is important to further explore the impact of other psychological factors on eating disordered behaviours such as body image, mood, depression or motivation, among others; due to the fact that they imply multifactorial aetiology of disorders. For example, some authors explained that it was clear that impression motivation was positively associated with eating disordered behaviours [28]. On the other hand, conducting clinical interviews is considered more accurate in the diagnosis of eating behaviours than self-administered questionnaires. No precise clinical diagnostic data were available to validate ED screening in the current study. Standard questionnaires are usually intended to screen for the risk of developing ED in the general population and might not necessarily be sensitive enough to detect the symptoms of these psychopathologies in athletes [23]. However, this fact is not an important limitation of our study, since the sample has also included a non-athlete population.

In the future, the use of other kinds of measurements, less subjected to the desirability bias, would help to discriminate whether the improvements in self-esteem and eating disorders in elite gymnasts are due to this bias or to preventing psychological treatments and trainings by coaches.

## 5. Conclusions

Previous literature has shown contradictory findings with regard to the prevalence of eating behaviours among young female athletes and non-athletes and in the prevalence rates of different sports disciplines, mainly by substantial inconsistencies in the methodology used. Despite athletes being at greater risk of eating disorders due to pressure to achieve a body composition that optimizes performance, the increased physical activity may contribute to a healthier attitude towards food and eating. In fact, the present study has shown a higher prevalence of DE attitudes in a non-athlete sample compared to the sports modalities studied. Screening for the female population as well as athletes at risk for EDs is important for the early detection and treatment efficacy. Coaches and teachers should target education regarding the risk factors of eating disorders, psychological well-being, nutrition, and body image. The role of adequate levels of self-esteem and perfectionism derived from a healthy educational context should be considered in preventing treatments, especially as protective factors of mass media influences when receiving restrictive aesthetic standard messages. Treating athletes and the young female population with EDs should be undertaken only by qualified health care professionals due to the severe physiological and psychological consequences of EDs, and to detect athletes at risk of the triad as early as possible. Further investigation is required to continue to understand eating behaviours in adolescents.

## Figures and Tables

**Table 1:** Mean and standard deviation (SD) of the main physical variables (age, weigh, height and BMI (Body-Mass Index).

	Condition	*N*	Scale Ranking	Mean	SD
**Age**	Gymnasts Footballers Control condition	40 40 40	15–25	16.60 17.98 17.13	2.62 3.42 2.16
**Weight**	Gymnasts Footballers Control condition	40 40 40	0–150 kg	47.69 58.86 55.74	5.93 8.61 7.09
**Height**	Gymnasts Footballers Control condition	40 40 40	60–200 cm	1.60 1.62 1.64	0.07 0.06 0.05
**BMI**	Gymnasts Footballers Control condition	40 40 40	Underweight 18.5–24.9 Overweight	18.72 22.45 20.73	1.54 2.93 2.32

**Table 2:** Multiple Linear Regression of factor associated with eating disorders (SCOFF).

Variables and Scales	Included Variables and Subscales	Β	*t*	*p*
Socio-demographic factors	Age	0.19	1.07	0.29
	Level of Studies	−0.14	−0.83	0.41
	Personal Background	−0.02	−0.22	0.83
	Kind of sport/control	−0.02	−0.22	0.82
Body weight status	Weight	1.28	1.41	0.16
	Height	−0.56	−1.32	0.19
	BMI	−0.90	−1.32	0.19
Self-Esteem	Self-Esteem Global	−0.22	−1.61	0.11
	Self-Esteem Positive	0.18	−1.61	0.11
	Self-Esteem Negative	0.14	1.41	0.16
Perfectionism	Self-Oriented Perfectionism	−0.11	−0.95	0.35
	Socially Prescribed Perfectionism	0.23	2.07	0.04
Anxiety	STAI-Estate	−0.23	−1.89	0.06
	STAI-Trait	0.21	1.73	0.09

**Table 3:** Spearman’s correlations between the main variables.

	BMI	RSE Global	CAPS Sub1	CAPS Sub2	STAI-S	STAI-T	EAT Sub1	EAT Sub2	EAT Sub3	SCOFF
Weight	0.90 **	−0.21 *	−0.13	0.001	0.06	−0.03	0.03	−0.10	−0.04	0.17
BMI		−0.13	−0.13	0.006	0.09	−0.03	0.07	−0.15	−0.12	0.10
RSE Global			0.24 **	−0.23 *	−0.39 **	−0.33 **	−0.17	−0.19 *	−0.01	−0.22 *
CAP Sub1				0.37 **	0.22 *	0.13	0.05	−0.16	−0.03	−0.11
CAP Sub2					0.34 **	0.32 **	0.015	0.06	−0.02	0.16
STAI-S						0.57 **	0.32 **	0.003	0.03	0.03
STAI-T							0.19 *	0.19 *	−0.04	0.14
EAT Sub1								0.35 *	0.18 *	0.12
EAT Sub2									0.18	0.11
EAT Sub3										0.09

** The correlation is significant at level 0.01 (bilateral) * The correlation is significant at level 0.05 (bilateral).

**Table 4:** Kruskal–Wallis test of the main variables of the study with group as factor.

	BMI	RSE G.	RSE+	RSE−	CAPS Sub1	CAPS Sub2	STAI-E	STAI-T	EAT Sub1	EAT Sub2	EAT Sub3	SCOFF
χ^2^	33.86	29.05	10.99	6.3	11.34	0.39	5.66	4.63	4.9	0.89	3.02	7.9
*p*	0.00	0.00	0.00	0.04	0.00	0.83	0.05	0.09	0.08	0.64	0.22	0.02

RSE G.: RSE Global; RSE+: RSE positive; RSE−: RESE negative.

**Table 5:** Mann–Whitney test between the three conditions (Gymnasts, Footballers and Control Condition).

	Scale Ranking	Condition	*N*	M	SD	Comparisons	χ^2^	*p*
**BMI**	Underweight 18.5–24.9 Overweight	Gymnasts	40	18.72	1.54	Gymnasts-Footballers	248	0.00
		Footballers	40	22.45	2.93	Footballers-Control Condition	512.5	0.00
		Control condition	40	20.73	2.32	Gymnasts-Control Condition	393	0.00
**RSE Global**	10–40	Gymnasts	40	33.73	4.14	Gymnasts-Footballers	526	0.00
		Footballers	40	31.33	4.35	Footballers-Control Condition	492	0.00
		Control condition	40	28.28	4.02	Gymnasts-Control Condition	256	0.00
**RSE Positive**	5–20	Gymnasts	40	33.73	2.07	Gymnasts-Footballers	656.5	0.16
		Footballers	40	31.33	2.51	Footballers-Control Condition	607	0.06
		Control condition	40	28.28	2.6	Gymnasts-Control Condition	458.5	0.00
**RSE Negative**	5–20	Gymnasts	40	13.15	2.19	Gymnasts-Footballers	566	0.02
		Footballers	40	12.1	2.35	Footballers-Control Condition	778	0.83
		Control condition	40	12.25	2.54	Gymnasts-Control Condition	589.5	0.04
**CAPS Subscale 1**	1–60	Gymnasts	40	38.28	7.25	Gymnasts-Footballers	699	0.33
		Footballers	40	36.58	7.33	Footballers-Control Condition	547	0.02
		Control condition	40	32.83	7.1	Gymnasts-Control Condition	471	0.00
**CAPS Subscale 2**	1–50	Gymnasts	40	25.48	8.61	Gymnasts-Footballers	749	0.62
		Footballers	40	26.2	8.64	Footballers-Control Condition	788	0.91
		Control condition	40	26.33	8.14	Gymnasts-Control Condition	740	0.56
**STAI-S**	0–60	Gymnasts	40	32.45	7.51	Gymnasts-Footballers	579	0.03
		Footballers	40	37.2	9.29	Footballers-Control Condition	773.5	0.8
		Control condition	40	37.35	11.46	Gymnasts-Control Condition	596.5	0.05
**STAI-T**	0–60	Gymnasts	40	32.85	6.89	Gymnasts-Footballers	613	0.07
		Footballers	40	35.83	8.07	Footballers-Control Condition	777.5	0.83
		Control condition	40	36.8	2.32	Gymnasts-Control Condition	602.5	0.06
**EAT Sub 1**	0–81	Gymnasts	40	5.13	9.92	Gymnasts-Footballers	639.5	0.12
		Footballers	40	8.2	10.03	Footballers-Control Condition	738	0.55
		Control condition	40	9.58	10.39	Gymnasts-Control Condition	580	0.03
**EAT Sub 2**	0–24	Gymnasts	40	1.85	2.41	Gymnasts-Footballers	765	0.72
		Footballers	40	1.73	2.31	Footballers-Control Condition	708.5	0.34
		Control condition	40	2.95	4.46	Gymnasts-Control Condition	746.5	0.59
**EAT Sub 3**	0–18	Gymnasts	40	0.68	1.49	Gymnasts-Footballers	703.5	0.17
		Footballers	40	0.33	0.92	Footballers-Control Condition	678	0.09
		Control condition	40	1.5	3.7	Gymnasts-Control Condition	767	0.68
**SCOFF**	0–5	Gymnasts	40	0.15	0.58	Gymnasts-Footballers	723	0.2
		Footballers	40	0.28	0.72	Footballers-Control Condition	675	0.11
		Control condition	40	0.55	0.93	Gymnasts-Control Condition	604	0.00

## References

[1] Conviser J.H., Tierney A.S., Nickols R. (2018). Essentials for best practice: Treatment approaches for athletes with eating disorders. J. Clin. Sport Psychol..

[2] Joy E., Kussman A., Nattiv A. (2016). 2016 update on eating disorders in athletes: A comprehensive narrative review with a focus on clinical assessment and management. Br. J. Sports Med..

[3] American Psychiatric Association (2013). Diagnostic and Statistical Manual of Mental Disorders.

[4] Arthur-Cameselle J., Sossin K., Quatromoni P. (2017). A qualitative analysis of factors related to eating disorder onset in female collegiate athletes and non-athletes. Eat. Disord..

[5] Knapp J., Aerni G., Anderson J. (2014). Eating disorders in female athletes: Use of screening tools. Curr. Sports Med. Rep..

[6] Byrne S., McLean N. (2001). Eating disorders in athletes: A review of the literature. J. Sci. Med. Sport.

[7] Smolak L., Murnen S.K., Ruble A.E. (2000). Female athletes and eating problems: A meta-analysis. Int. J. Eat. Disord..

[8] Sundgot-Borgen J., Torstveit M.K. (2010). Aspects of disordered eating continuum in elite high-intensity sports. Scand. J. Med. Sci. Sports.

[9] Taub D.E., Blinde E.M. (1992). Eating disorders among adolescent female athletes: Influence of athletic participation and sport team membership. Adolescence.

[10] Scott C.L., Haycraft E., Plateau C.R. (2019). Teammate influences on the eating attitudes and behaviours of athletes: A systematic review. Psychol. Sport Exerc..

[11] Petrie T.A., Greenleaf C.A., Tenenbaum G., Eklund R.C. (2007). Eating disorders in sport. From theory to research to intervention. Handbook of Sport Psychology.

[12] Krentz E., Warschburger P. (2011). Sports-related correlates of disordered eating in aesthetic sports. Psychol. Sport Exerc..

[13] Rosa-Caldwell M.E., Todden C., Caldwell A.R., Breithaupt L.E. (2018). Confidence in eating disorder knowledge does not predict actual knowledge in collegiate female athletes. PeerJ.

[14] Keel P.K., Forney K.J. (2013). Psychosocial risk factors for eating disorders. Int. J. Eat. Disord..

[15] Kristjánsdóttir H., Siguroardóttir P., Jónsdóttir S., Porsteinsdóttir G., Saavedra J. (2019). Body image concern and eating disorder symptoms among elite Icelandic athletes. Int. J. Environ. Res. Public Health.

[16] Deering S. (2001). Eating disorders: Recognition, evaluation, and implications for obstretrician/gynecologists. Prim. Care Update Ob. Gyns..

[17] El Ghoch M., Soave F., Calugi S., Dalle Grave R. (2013). Eating disorders, physical fitness and sport performance: A systematic review. Nutrients.

[18] Coelho G.M., Soares E.A., Ribeiro B.G. (2010). Are female athletes at increased risk for disordered eating and its complications?. Appetite.

[19] Sundgot-Borgen J., Torstveit M.K. (2004). Prevalence of eating disorders in elite athletes is higher than in the general population. Clin. J. Sport Med..

[20] Thiemann P., Legenbauer T., Vocks S., Platen P., Auyeung B., Herpertz S. (2015). Eating disorders and their putative risk factors among female German professional athletes. Eur. Eat. Disord. Rev..

[21] Kerr G., Berman E., De Souza M.J. (2006). Disordered eating in women’s gymnastics: Perspectives of athletes, coaches, parents, and judges. J. Appl. Sport Psychol..

[22] Sanford-Martens T.C., Megan Davidson M., Yakushko O.F., Martens M., Hinton P., Beck N. (2005). Clinical and subclinical eating disorders: An examination of collegiate athletes. J. Appl. Sport Psychol..

[23] Fortes L.S., Kakeshita I.S., Almeida S.S., Gomes A.R., Ferreira M.E.C. (2014). Eating behaviours in youths: A comparison between female and male athletes and non-athletes. Scand. J. Med. Sci. Sports.

[24] Michou M., Costarelli V. (2011). Disordered Eating Attitudes in relation to anxiety levels, self-steem and body image in female basketball players. J. Exerc. Sci. Fit..

[25] Rosendahl J., Bormann B., Aschenbrenner K., Aschenbrenner F., Strauss B. (2008). Dieting and disordered eating in German high school athletes and non-athletes. Scand. J. Med. Sci. Sports.

[26] Arcelus J., Witcomb G., Mitchell A. (2014). Prevalence of eating disorders amongst dancers: A systematic review and meta-analysis. Eur. Eat. Disord. Rev..

[27] Petrie T.A., Greenleaf C.A., Reel J.J., Carter J.E. (2009). An examination of psychosocial correlates of eating disorders among female collegiate athletes. Res. Q. Exerc. Sport.

[28] Rui Gomes A., Martins C., Silva L. (2011). Eating disordered behaviours in Portuguese athletes: The influence of personal, sport, and psychological variables. Eur. Eat. Disord. Rev..

[29] Currie A. (2010). Sport and eating disorders-understanding and managing the risks. Asian J. Sports Med..

[30] Haase A.M., Prapavessis H., Owens R.G. (2002). Perfectionism, social physique anxiety and disordered eating: A comparison of male and female elite athletes. Psychol. Sport Exerc..

[31] Davey G.C.L., Chapman L. (2009). Disgust and eating disorder symptomatology in a non-clinical population: The role of trait anxiety and anxiety sensitivity. Clin. Psychol. Psychother..

[32] Stoeber J. (2011). The dual nature of perfectionism in sports: Relationships with emotion, motivation, and performance. Int. J. Sport Exerc. Psychol..

[33] Giacobbi P.R., Weinberg R.S. (2000). An examination of coping in sport: Individual trait anxiety differences and situational consistency. Sport Psychol..

[34] De Bruin K., Bakker F.C., Oudejans R. (2009). Achievement goal theory and disordered eating: Relationships of disordered eating with goal orientations and motivational climate in female gymnasts and dancers. Psychol. Sport. Exerc..

[35] Koivula N., Hassmén P., Fallby J. (2002). Self-esteem and perfectionism in elite athletes: Effects on competitive anxiety and self-confidence. Pers. Individ. Differ..

[36] Fairburn C.G., Cooper Z., Shafran R. (2003). Cognitive behaviour therapy for eating disorders: A transdiagnostic theory and treatment. Behav. Res. Ther..

[37] O’Dea J.A. (2009). Self perception score from zero to ten correlates well with standardized scales of adolescent self esteem, body dissatisfaction, eating disorders risk, depression, and anxiety. Int. J. Adolesc. Med. Health.

[38] Bardone-Cone A.M., Wonderlich S.A., Frost R.O., Bulik C.M., Mitchell J.E., Uppala S., Simonich H. (2007). Perfectionism and eating disorders: Current status and future directions. Clin. Psychol. Rev..

[39] Brockmeyer T., Holtforth M.G., Bents H., Kämmerer A., Herzog W., Friederich H.C. (2013). The thinner the better: Self-esteem and low body weight in anorexia nervosa. Clin. Psychol. Psychother..

[40] Bardone-Cone A.M., Abramson L.Y., Vohs K.D., Heatherton T.F., Joiner T.E. (2006). Predicting bulimic symptoms: An interactive model of self-efficacy, perfectionism, and perceived weight status. Behav. Res. Ther..

[41] Puttevils L., Vanderhasselt M.A., Vervaet M. (2019). Investigating transdiagnostic factors in eating disorders: Does self-esteem moderate the relationship between perfectionism and eating disorder symptoms?. Eur. Eat. Disord. Rev..

[42] Rosenberg M. (1965). Society and the Adolescent Self-Image.

[43] González F.L.A., Sigüenza Y.M., Solá I.B. (2000). Análisis de la dimensionalidad de la Escala de Autoestima de Rosenberg en una muestra de adolescentes valencianos. Rev. Psicol. Univ. Tarracon..

[44] Donaldson D., Spirito A., Farnett E. (2000). The role of perfectionism and depressive cognitions in understanding the hopelessness experienced by adolescent suicide attempters. Child Psychiatry Hum. Dev..

[45] Hewitt P.L., Flett G.L. (1991). Perfectionism in the self and social contexts: Conceptualization, assessment, and association with psychopathology. J. Pers. Soc. Psychol..

[46] Castro J., Gila A., Gual P., Lahortiga F., Saura B., Toro J. (2004). Perfectionism dimensions in children and adolescents with anorexia nervosa. J. Adolesc. Health.

[47] Spielberger C.D., Gorsuch R., Lushene R. (1970). Manual for the State-Trait Anxiety Inventory.

[48] Spielberger C.D., Gorsuch R.L., Lushene R. (1982). Manual del Cuestionario de Ansiedad Estado/Rasgo (STAI).

[49] Guillén-Riquelme A., Buela-Casal G. (2011). Actualización psicométrica y funcionamiento diferencial de los ítems en el State Trait Anxiety Inventory (STAI). Psicothema.

[50] Garner D.M., Garfinkel P.E. (1979). The Eating Attitudes Test: And index of the symptoms of anorexia nervosa. Psychol. Med..

[51] Castro J., Toro J., Salamero M., Guimerá E. (1991). The Eating Attitudes Test: Validation of the Spanish version. Psychol. Assess..

[52] Morgan J.F., Reid F., Lacey J.H. (1999). The SCOOF questionnaire: Assessment of a new screening tool for eating disorders. Br. Med. J..

[53] Herpertz-Dahlmann B., Wille N., Höllling H., Vloet T.D., Ravens-Sieberer U. (2008). Disordered eating behaviour and attitudes, associated psychopathology and health-related quality of life: Results of the BELLA study. Eur. Child Adolesc. Psychiatry.

[54] Hill L.S., Reid F., Morgan J.F., Lacey J.H. (2010). SCOFF, the development of an eating disorder screening questionnaire. Int. J. Eat. Disord..

[55] Currin L., Schmidt U. (2005). A critical analysis of the utility of an early intervention approach in the eating disorders. J. Ment. Health.

[56] Augestad L., Flanders W. (2002). Eating disorder behaviour in physically active Norwegian women. Scand. J. Med. Sci. Sports.

[57] Salas-Salvado J., Rubio M.A., Barbany M., Moreno B. (2007). SEEDO 2007 Consensus for the evaluation of overweight and obesity and the establishment of therapeutic intervention criteria. Med. Clin..

[58] Borrione P., Battaglia C., Di Cagno A. (2013). No risk of anorexia nervosa in young rhythmic gymnasts: What are the practical implications of what is already know?. J. Nutr. Disord. Ther..

[59] Toselli A.L., Villani S., Ferro A.M., Verri A., Cucurullo L., Marinoni A. (2005). Eating disorders and their correlates in high school adolescents of Northern Italy. Epidemiol. Psychiatr. Sci..

[60] Torstveit M.K., Rosenvinge J.H., Sundgot-Borgen J. (2008). Prevalence of eating disorders and predictive power of risk models in female athletes elite: A controlled study. Scand. J. Med. Sci. Sports.

[61] Thompson R.A., Trattner Sherman R. (2010). Eating Disorders in Sports.

[62] Stice E., Pressnell K., Spangler D. (2002). Risk factors for binge eating onset in adolescent girls: A 2-year prospective investigation. Health Psychol..

[63] Burrows A., Cooper M. (2002). Possible risk factors in the development of eating disorders in overweight pre-adolescent girls. Int. J. Obes..

[64] McClure A.C., Tanski S.E., Kingsbury J., Gerrard M., Sargent J.D. (2010). Characteristics associated with low self-esteem among US adolescents. Acad. Pediatr..

[65] Cruz-Sáez S., Pascual A., Wlodarczyk A., Echeburúa E. (2018). The effect of body dissatisfaction on disordered eating: The mediating role of self-esteem and negative affect in male and female adolescents. J. Health Psychol..

[66] Striegel-Moore R.H., Dohm F.A., Kraemer H.C., Taylor C.B., Daniels S., Crawford P.B., Schreiber G.B. (2003). Eating disorders in white and black women. Am. J. Psychiatry.

[67] Jauregui-Lobera I., Romero Candau J., Montaña Gonzalez M.T., Morales Millán M.T., Sánchez N., León Lozano P. (2009). Analysis of eating attitudes in a simple of adolescents from Sevilla. Med. Clin..

[68] Luck A.J., Morgan J.F., Reid F., O’Brien A., Brunton J., Price C., Perry L., Lacey J.H. (2002). The SCOFF questionnaire and clinical interview for eating disorders in general practice: Comparative study. Br. Med. J..

[69] Tavolacci M.P., Grigioni S., Richard L., Meyrignac G., Déchelotte P., Ladner J. (2015). Eating disorders and associated health risks among university students. J. Nutr. Educ. Behav..

[70] Fragkos K.C., Frangos C.C. (2013). Assesing eating disorder risk: The pivotal role of achievement anxiety, depression and female gender in non-clinical samples. Nutrients.

[71] Martinsen M., Brantland-Sanda S., Eriksson A.K., Sundgot-Borgen J. (2010). Dieting to win or to thin? A study of dieting and disordered eating among adolescent elite athletes and non-athlete controls. Br. J. Sports Med..

[72] Sundgot-Borgen J. (1993). Prevalence of eating disorders in elite female athletes. Int. J. Sport Nutr..

[73] Fortes L.S., Ferreira M.E.C. (2011). Comparison of body dissatisfaction and inappropriate eating behaviour in adolescent athletes of different sports. Braz. J. Phys. Educ. Sport.

[74] Coelho G.M., De Farias M.L., De Mendonca L.M., De Mello D.B., Lanzillotti H.S., Ribeiro B.G., Soares E.A. (2013). The prevalence of disordered eating and possible health consequences in adolescent female tennis players from Rio de Janeiro, Brazil. Appetite.

[75] Costarelli V., Stamou D. (2009). Emotional Intelligence, body image and disordered eating attitudes in combat sport athletes. J. Exerc. Sci. Fit..

[76] De Bruin K., Oudejans R., Bakker F.C. (2007). Dieting and body image in aesthetic sports: A comparison of Dutch female gymnasts and nonaesthetic sport participants. Psychol. Sport Exerc..

[77] Bar R.J., Cassin S.E., Dionne M.M. (2016). Eating disorder prevention initiatives for athletes: A review. Eur. J. Sport Sci..

[78] Coelho G.M., Da Silva Gomes A.I., Ribeiro B.G., Soares E.A. (2014). Prevention of eating disorders in female athletes. Review. J. Sports Med..

[79] Krentz E., Warschburger P. (2013). A longitudinal investigations of sports-related risk factors for disordered eating in aesthetic sports. Scand. J. Med. Sci. Sports.

[80] Petrie T.A. (1993). Disordered eating in female collegiate gymnasts: Prevalence and personality/attitudinal correlates. J. Sport Exerc. Psychol..

[81] Sanborn C., Horea M., Simers B., Dieringer K. (2000). Disordered eating and the female athlete triad. Clin. Sports Med..

[82] Sundgot-Borgen J., Meyer N.L., Lohman T.G., Ackland T.R., Maughan R.J., Stewart A.D., Muller W. (2013). How to minimize the health risks to athletes who compete in weight-sensitive sports review and position statement on behalf of the Ad Hoc Research Working Group on Body Composition, Health and Performance, under the auspices of the IOC Medical Commission. Br. J. Sports Med..

[83] Cook B.J., Hausenblas H.A. (2008). The role of exercise dependence for the relationship between exercise behaviour and eating pathology: Mediator or moderator?. J. Health Psychol..

[84] Haase A.M. (2011). Weight perception in female athletes: Associations with disordered eating correlates and behaviour. Eat. Behav..

[85] Vardar E., Vardar S.A., Kurt C. (2007). Anxiety of young female athletes with disordered eating behaviours. Eat. Behav..

[86] Pollice C., Kaye W.H., Greeno C.G., Weltzin T.E. (1997). Relationship of depression, anxiety, and obsessionality to state of illness in anorexia nervosa. Int. J. Eat. Disord..

[87] Holm-Denoma J.M., Scaringi V., Gordon K.H., Van Orden K., Joiner T.E. (2009). Eating disorder symptoms among undergraduate varsity athletes, club athletes, independent exercisers, and nonexercisers. Int. J. Eat. Disord..

[88] Frost R.O., Marten P., Lahart C., Rosenblate R. (1990). The dimensions of perfectionism. Cogn. Ther. Res..

[89] Davis C., Claridge G., Fox J. (2000). Not just a pretty face: Physical attractiveness and perfectionism in the risk for eating disorders. Int. J. Eat. Disord..

[90] Longbottom J.L., Grove J.R., Dimmock J.A. (2012). Trait perfectionism, self-determination, and self-presentation processes in relation to exercise behaviour. Psychol. Sport Exerc..

[91] Granillo T., Jones-Rodriguez G., Carvajal S.C. (2005). Prevalence of eating disorders in Latina adolescents: Associations with substance use and other correlates. J. Adolesc. Health.

[92] Armstrong S., Oomen-Early J. (2009). Social connectedness, self-esteem, and depression symptomatology among collegiate athletes versus nonathletes. J. Am. Coll. Health.

[93] Kantanista A., Glapa A., Banio A., Firek W., Ingarden A., Malchrowicz-Mośko E., Markiewicz P., Płoszaj K., Ingarden M., Maćkowiak Z. (2018). Body image of highly trained female athletes engaged in different types of sport. BioMed Res. Int..

[94] Naeimi A.F., Haghighian H.K., Gargari B.P., Alizadeh M., Rouzitalab T. (2016). Eating disorders risk and its relation to self-esteem and body image in Iranian university students of medical sciences. Eat. Weight Disord..

